# Supplementary material for: Individual retrotransposon integrants are differentially controlled by KZFP/KAP1-dependent histone methylation, DNA methylation and TET-mediated hydroxymethylation in naïve embryonic stem cells
Source: Epigenetics Chromatin. 2018 Feb 26;11:7. doi: 10.1186/s13072-018-0177-1 (PMC6389204; doi:10.1186/s13072-018-0177-1)
Supplement: Supplementary file 11 — Additional file 11. Pattern analysis. [file 13072_2018_177_MOESM11_ESM.zip › Patterns analysis/DataTables/extensions/TableTools/examples/select_single.html]

TableTools example - Row selection - single row select


# TableTools example Row selection - single row select

As well as providing a button toolbar, TableTools provides everything needed to have selectable rows
in the table. TableTools has four row selection modes of operation:

- `none` - Default, where no user row selection options are available
- `single` - A single row can be selected
- `multi` - Multiple rows can be selected simply by clicking on the rows
- `os` - Operating System like selection where you can use the shift and ctrl / cmd
  keys on your keyboard to add / remove rows from the selection.

This example shows the `single` select option.

| Name | Position | Office | Age | Start date | Salary |
| --- | --- | --- | --- | --- | --- |
| Name | Position | Office | Age | Start date | Salary |
| --- | --- | --- | --- | --- | --- |
| Tiger Nixon | System Architect | Edinburgh | 61 | 2011/04/25 | $320,800 |
| Garrett Winters | Accountant | Tokyo | 63 | 2011/07/25 | $170,750 |
| Ashton Cox | Junior Technical Author | San Francisco | 66 | 2009/01/12 | $86,000 |
| Cedric Kelly | Senior Javascript Developer | Edinburgh | 22 | 2012/03/29 | $433,060 |
| Airi Satou | Accountant | Tokyo | 33 | 2008/11/28 | $162,700 |
| Brielle Williamson | Integration Specialist | New York | 61 | 2012/12/02 | $372,000 |
| Herrod Chandler | Sales Assistant | San Francisco | 59 | 2012/08/06 | $137,500 |
| Rhona Davidson | Integration Specialist | Tokyo | 55 | 2010/10/14 | $327,900 |
| Colleen Hurst | Javascript Developer | San Francisco | 39 | 2009/09/15 | $205,500 |
| Sonya Frost | Software Engineer | Edinburgh | 23 | 2008/12/13 | $103,600 |
| Jena Gaines | Office Manager | London | 30 | 2008/12/19 | $90,560 |
| Quinn Flynn | Support Lead | Edinburgh | 22 | 2013/03/03 | $342,000 |
| Charde Marshall | Regional Director | San Francisco | 36 | 2008/10/16 | $470,600 |
| Haley Kennedy | Senior Marketing Designer | London | 43 | 2012/12/18 | $313,500 |
| Tatyana Fitzpatrick | Regional Director | London | 19 | 2010/03/17 | $385,750 |
| Michael Silva | Marketing Designer | London | 66 | 2012/11/27 | $198,500 |
| Paul Byrd | Chief Financial Officer (CFO) | New York | 64 | 2010/06/09 | $725,000 |
| Gloria Little | Systems Administrator | New York | 59 | 2009/04/10 | $237,500 |
| Bradley Greer | Software Engineer | London | 41 | 2012/10/13 | $132,000 |
| Dai Rios | Personnel Lead | Edinburgh | 35 | 2012/09/26 | $217,500 |
| Jenette Caldwell | Development Lead | New York | 30 | 2011/09/03 | $345,000 |
| Yuri Berry | Chief Marketing Officer (CMO) | New York | 40 | 2009/06/25 | $675,000 |
| Caesar Vance | Pre-Sales Support | New York | 21 | 2011/12/12 | $106,450 |
| Doris Wilder | Sales Assistant | Sidney | 23 | 2010/09/20 | $85,600 |
| Angelica Ramos | Chief Executive Officer (CEO) | London | 47 | 2009/10/09 | $1,200,000 |
| Gavin Joyce | Developer | Edinburgh | 42 | 2010/12/22 | $92,575 |
| Jennifer Chang | Regional Director | Singapore | 28 | 2010/11/14 | $357,650 |
| Brenden Wagner | Software Engineer | San Francisco | 28 | 2011/06/07 | $206,850 |
| Fiona Green | Chief Operating Officer (COO) | San Francisco | 48 | 2010/03/11 | $850,000 |
| Shou Itou | Regional Marketing | Tokyo | 20 | 2011/08/14 | $163,000 |
| Michelle House | Integration Specialist | Sidney | 37 | 2011/06/02 | $95,400 |
| Suki Burks | Developer | London | 53 | 2009/10/22 | $114,500 |
| Prescott Bartlett | Technical Author | London | 27 | 2011/05/07 | $145,000 |
| Gavin Cortez | Team Leader | San Francisco | 22 | 2008/10/26 | $235,500 |
| Martena Mccray | Post-Sales support | Edinburgh | 46 | 2011/03/09 | $324,050 |
| Unity Butler | Marketing Designer | San Francisco | 47 | 2009/12/09 | $85,675 |
| Howard Hatfield | Office Manager | San Francisco | 51 | 2008/12/16 | $164,500 |
| Hope Fuentes | Secretary | San Francisco | 41 | 2010/02/12 | $109,850 |
| Vivian Harrell | Financial Controller | San Francisco | 62 | 2009/02/14 | $452,500 |
| Timothy Mooney | Office Manager | London | 37 | 2008/12/11 | $136,200 |
| Jackson Bradshaw | Director | New York | 65 | 2008/09/26 | $645,750 |
| Olivia Liang | Support Engineer | Singapore | 64 | 2011/02/03 | $234,500 |
| Bruno Nash | Software Engineer | London | 38 | 2011/05/03 | $163,500 |
| Sakura Yamamoto | Support Engineer | Tokyo | 37 | 2009/08/19 | $139,575 |
| Thor Walton | Developer | New York | 61 | 2013/08/11 | $98,540 |
| Finn Camacho | Support Engineer | San Francisco | 47 | 2009/07/07 | $87,500 |
| Serge Baldwin | Data Coordinator | Singapore | 64 | 2012/04/09 | $138,575 |
| Zenaida Frank | Software Engineer | New York | 63 | 2010/01/04 | $125,250 |
| Zorita Serrano | Software Engineer | San Francisco | 56 | 2012/06/01 | $115,000 |
| Jennifer Acosta | Junior Javascript Developer | Edinburgh | 43 | 2013/02/01 | $75,650 |
| Cara Stevens | Sales Assistant | New York | 46 | 2011/12/06 | $145,600 |
| Hermione Butler | Regional Director | London | 47 | 2011/03/21 | $356,250 |
| Lael Greer | Systems Administrator | London | 21 | 2009/02/27 | $103,500 |
| Jonas Alexander | Developer | San Francisco | 30 | 2010/07/14 | $86,500 |
| Shad Decker | Regional Director | Edinburgh | 51 | 2008/11/13 | $183,000 |
| Michael Bruce | Javascript Developer | Singapore | 29 | 2011/06/27 | $183,000 |
| Donna Snider | Customer Support | New York | 27 | 2011/01/25 | $112,000 |

- Javascript
- HTML
- CSS
- Ajax
- Server-side script

The Javascript shown below is used to initialise the table shown in this
example:

`$(document).ready(function() {
$('#example').DataTable( {
dom: 'T<"clear">lfrtip',
tableTools: {
"sRowSelect": "single"
}
} );
} );`

In addition to the above code, the following Javascript library files are loaded for use in this
example:

- ../../../media/js/jquery.js
- ../../../media/js/jquery.dataTables.js
- ../js/dataTables.tableTools.js

The HTML shown below is the raw HTML table element, before it has been enhanced by
DataTables:

This example uses a little bit of additional CSS beyond what is loaded from the library
files (below), in order to correctly display the table. The additional CSS used is shown
below:

The following CSS library files are loaded for use in this example to provide the styling of the
table:

- ../../../media/css/jquery.dataTables.css
- ../css/dataTables.tableTools.css

This table loads data by Ajax. The latest data that has been loaded is shown below. This data
will update automatically as any additional data is loaded.

The script used to perform the server-side processing for this table is shown below. Please note
that this is just an example script using PHP. Server-side processing scripts can be written in any
language, using the protocol described in the
DataTables documentation.

## Other examples

### Examples

- Basic initialisation
- Plug-in button types
- Bootstrap styling
- Setting the SWF path
- Initialisation with `new`
- Defaults
- Row selection - single row
  select
- Row selection - multi-row select
- Row selection - operating system style
- Row selection - row selector on specific cells
- Multiple tables
- Multiple toolbars
- Button collections
- Custom button text
- Button arrangement
- Ajax loaded data
- PDF message
- jQuery UI styling

Please refer to the DataTables documentation for full
information about its API properties and methods.  
Additionally, there are a wide range of extras and
plug-ins which extend the capabilities of
DataTables.

DataTables designed and created by SpryMedia Ltd © 2007-2014  
DataTables is licensed under the MIT license.
